# Weak Evidence of Regeneration Habitat but Strong Evidence of Regeneration Niche for a Leguminous Shrub

**DOI:** 10.1371/journal.pone.0130886

**Published:** 2015-06-22

**Authors:** Florian Delerue, Maya Gonzalez, Richard Michalet, Sylvain Pellerin, Laurent Augusto

**Affiliations:** 1 INRA, UMR 1391 ISPA, Villenave d’Ornon F-33140, France; 2 Bordeaux Science Agro, UMR 1391 ISPA, Gradignan F-33883, France; 3 Bordeaux INP, G&E, EA 4592, F-33600, Pessac, France; 4 Univ. Bordeaux Montaigne, G&E, EA 4592, F-33600, Pessac, France; 5 Université de Bordeaux, UMR 5805 EPOC, F-33405 Talence, France; University of Tartu, ESTONIA

## Abstract

The identification of an ecological niche specific to the regeneration phase has mobilised significant attention. However, the importance of the regeneration niche concept remains unclear. Our main objective was to study the existence of such a regeneration niche for a leguminous shrub, *Ulex europaeus*. This study was carried out in southwest France in the context of water and nutrient stresses (mainly phosphorus limitation) due to the presence of nutrient-poor sandy soils. We analysed the regeneration of the species from the germination of seeds and emergence of new seedlings until the seedlings reached young shrub size. Our design included a P fertilisation treatment. We also investigated microsite characteristics (micro-topography and vegetation development) as they can interact with meteorological conditions and determine water availability for seeds and seedlings. We found that P availability controlled seedling growth and the time necessary to reach young shrub size. Water availability appeared to impact the species germination and seedlings survival. We also found that P and water availability depended on the interactions between microsite characteristics and climatic variations. Finally we found evidence that P and water availability are important ecological factors shaping the regeneration niche of the species, but we found weak evidence that any microsite would be appropriate for the regeneration of the species in the long term. Future studies regarding regeneration niches need to distinguish more clearly the ecological factors important for regeneration (the regeneration niche *per se*) and the physical world where the seedlings appear and develop (the regeneration habitat).

## Introduction

The regeneration phase is of prime importance in the life history of woody plants. It determines species population dynamics, and in turn plant community assemblages. Following the pioneer work of Grubb [1], the identification of an ecological niche specific to the regeneration phase (i.e. the ‘regeneration niche’) has mobilised significant attention, particularly in forest systems [2,3]. However, conclusions from many field studies are equivocal regarding the existence of regeneration niches, that is to say the existence of contrasting ecological drivers of regeneration for different species, so the existence of the regeneration niche remains an on-going debate.

Our objective was to contribute to this debate by exploring *in situ* the existence of such a regeneration niche for a woody species, *Ulex europaeus L*. (hereafter referred to as gorse). We made this choice because gorse is spontaneously present in a one million hectare area of south-western France, the Landes de Gascogne forest, which is characterized by acidic sandy soils [4], an extreme P deficiency for seedlings [5] and trees [6], and intense water stress during the summer period [7]. Regarding these severe environmental conditions, we assumed that water and P limitations influence the regeneration of the species, and that water and P availabilities might be important ecological factors shaping its regeneration niche. Moreover, P-limitation is known to strongly affect legumes [8,9], and especially their seedlings [10]. Choosing *Ulex europaeus*—a legume showing a positive response of its mature individuals to P fertilization [11]—was thus appropriate for the purpose of our study.

In addition to these abiotic factors, vegetation that is already present when a new seedling appears may have contrasting effects on its survival. In cases of severe abiotic stress, facilitation is likely to be more important than competition according to the Stress Gradient Hypothesis [12,13]. In our study region, low rainfall and water stress is common during the summer [7]. Under these conditions, facilitation through limitation of water stress has been demonstrated in the region [14] as for *Ulex parviflorus* seedlings in Mediterranean climates [15]. Moreover, Le Bagousse *et al*. [14] have shown that fertilisation increases the development of nurse plants. It leads to higher facilitation in cases of water stress and higher competition when water stress is released. Based on these results, we chose to pay a particular attention to biotic interactions between gorse seedlings and the surrounding plants.

Our study was carried out in a clear-cut stand of maritime pine forest in southwest France for two consecutive years (from 2010 to 2012). To study the regeneration of the species, we focused on the recruitment stage which includes all stages from germination to the successful installation of a new individual (i.e. when the risk of death decreases sharply; see criteria in S1 File). Therefore, emergence (the appearance of new seedlings after seed germination), survival and growth of seedlings were monitored during the two years of study. Our experimental design included a P fertilisation treatment, as this element could influence seedling development and biological nitrogen fixation. Because plant-plant interactions, and plant-soil interactions, may change sharply over a period of a few weeks [16], which in turn influence the fate of seedlings [17], an appropriate scale of analysis was required to monitor gorse regeneration. That is why we carried out our monitoring regularly, approximately every 50 days. Similarly, we carried out our investigations at two spatial scales: a coarse 1 m^2^ scale and a finer 0.01 m^2^ scale. Indeed, it has been shown that abiotic ecological factors that can impact gorse regeneration (particularly water and P availability) can vary at fine spatial scales (e.g. a centimetric scale [18]). We used the former scale as it corresponds to a classical approach in the literature regarding regeneration niches (e.g. [19,20]). We used the latter scale to capture fine environmental variations that could influence gorse regeneration as shown in a few other studies [21,22]. We paid particular attention to topographic variations and vegetation development surrounding the seedlings at both scales because they could influence water and P availabilities for seedlings.

## Materials and Methods

### Study area

The study was carried out from May 2010 to April 2012 in the planted maritime pine forest of the 'Landes de Gascogne' plain. In the region, soils are acidic podzols, composed of more than 90% sands and with very low nutrient contents [4]. Plant growth in this region has been shown to be mainly limited by low phosphorus availability [6]. The climate is temperate oceanic, with an annual precipitation of about 900 mm (mean value of the last 30 years; source INRA-Aquitaine). Rainfall is distributed throughout the year (an average of approximately 80 mm month^-1^ from autumn to spring). In summer, rainfall is generally only slightly lower (approx. 55 mm month^-1^) but periods of particularly low rainfall and important water stress occur in some years [7].

### The model species

This field study focused on common gorse, an evergreen leguminous shrub native from the European Atlantic coast. Adult plants are light-demanding, with the species being most abundant after a disturbance at the pioneer phase of the forest cycle. Its fecundity is high in full light conditions [23] and seeds are dispersed locally, rarely reaching distances farther than 2.5 m from the parent plant [24]. Dispersed seeds form a persistent seed bank and seeds located in the first five centimetres of the topsoil can germinate and emerge at the surface [25]. Germination is opportunistic and can occur throughout the year, except in winter, and is favoured by warm wet periods [26]. Young seedlings are sensitive to interspecific competition [25] and contrary to adult individuals, seedlings show strong shade-tolerance [27].

### Experimental design

#### The stand and the treatments

The study was set up after a clear cut and soil ploughing that occurred in November 2009 in a pine (*Pinus pinaster*) stand, 225 m in length and 30 m in width (44.73°N, 0.73°W). The stand is under the authority of the French National Institute of Agricultural Reseach (INRA) who gave permission to conduct the study. Sampling of the seed bank from the topsoil (0–5 cm) showed that its mean density was approximately 28±6 seeds.m^-2^ (90 samples were collected throughout the stand; unpublished data). The seed bank was low compared to densities measured in the region [28]. After the clear cut, colonisation of the stand by spontaneous vegetation occurred during the course of the study and was monitored using dedicated allometric relationships [29]. Dominant species were herbaceous, mainly purple moor grass (*Molinia caerulea*; 143±10 g m^-2^), constituting 90% of the aboveground vegetation biomass at the end of the first year of the study.

Two treatments (P fertilisation and sowing of gorse seeds) were applied in the stand after the clear cut and repeated in three blocks that split the stand lengthwise. P fertilisation was done because of the potential importance of P availability for the leguminous species regeneration. Sowing ensured that regeneration was not limited by seed availability. Each treatment had two levels: *1)* fertilisation with inorganic phosphate (0 or 160 kg-P_2_O_5_ ha^-1^); *2)* sown gorse seeds (0 or 100 seeds m^-2^). The treatments were randomly crossed in each block giving 4 combinations (unfertilised with sowing, unfertilised without sowing, fertilised with sowing, fertilised without sowing). The sowing was done by hand using a mixture of seeds and white sand to control sowing homogeneity and then seeds were buried slightly with a harrow.

#### The responses of the studied species

In many studies of regeneration of woody plants, the distinction between seedlings and young shrubs or trees relies on a size threshold which appears somewhat arbitrary (e.g. [30,31]). Clear determination of the end of the regenerative phase can be a critical issue when studying the regeneration niche. Thus we tried to determine an objective criterion of recruitment success based upon: *1)* a size threshold from which the number of deaths observed was almost zero (see Figure A, panel a, in S1 File); *2)* a size threshold from which growth rates of gorse plants increased sharply (Figure A, panel b, in S1 File); and *3)* the measurement of the mean height of the herbaceous layer in the stand, as an indicator of the height that gorse plants had to reach, to get a better access to light. Based on consistent results, we finally assumed that plants which reached 12 cm in height became were successfully recruited and became young shrubs (see S1 File).

Seedling emergence, survival, growth and recruitment success were monitored for two years every 50 ± 5 days, except during winter, with no survey from the beginning of December to the end of March, thus giving a total of 12 censuses. This monitoring was done at two different scales: the coarse-grained scale consisted of 72 quadrats of 1 m^2^ placed within the stand, giving 24 quadrats per block. In each block, the 4 combinations of the treatments received 6 quadrats. Distribution of the quadrats within the different combinations was semi-systematic to ensure the presence of quadrats throughout the whole stand surface. At this scale, the main responses of the model species recorded were: the number of new seedlings that emerged (discrete variable, 0 to n), the proportion of dead seedlings (between 0 and 1), and the proportion of seedlings successfully recruited (i.e. that reached the size threshold and became young shrubs, between 0 and 1).

We considered that each quadrat was composed of 100 microsites formed by square cells, each of 0.01 m^2^. Each cell (with sides of 10 cm) had two coordinates, x and y, from 1 to 10, the cells [1,1] and [10,10] being at the South West and North East corner of the quadrat respectively. This microsite scale formed the fine-grained scale of the study. At this scale the main responses of the model species recorded were: emergence of a new seedling in the microsite (binary response, 0 or 1), death (absence of a living seedling that had been observed before, binary response, 0 or 1), size (length of the longest shoot in cm, continuous variable), and recruitment success (binary response, 0 or 1). In the rare cases when 2 seedlings were present in the same cell, their positions in the cell were recorded to avoid confusion between them. Characterisation of these microsites (i.e. classes of micro-topography, classes of vegetation index, see below) required the knowledge of the whole neighbourhood of the microsites. Thus, microsites at the edge of the quadrats were excluded from all analyses because their neighbourhoods were incompletely recorded. In our design, the total number of microsites was 4608 (64 central microsites quadrat^-1^ x 72 quadrats).

#### P availability and symbiotic nitrogen fixation by gorse seedlings

Whatever the scale of analysis, we assumed that quadrats and microsites within quadrats that were distributed in the P fertilisation treatments were situated in areas with higher P availability. An important response to P fertilisation for legumes is the potential improvement of biological nitrogen fixation. Thus each year, an additional sample of seedlings, that were located outside the 72 observed quadrats, were harvested to measure the proportion of fixed nitrogen derived from the atmosphere (%N_dfa_) in their tissues. We compared the %Ndfa of gorse seedlings from fertilised and unfertilised areas. An additional harvest of gorse seedlings was also made in 2013, after the ending of the main study, and with the same objective. %N_dfa_ determination was based on the ^15^N isotopic dilution method [32], and by using a homogenous spray of labelled ^15^NH_4_Cl solution at the beginning of the study after soil preparation. More details about the ^15^N isotopic dilution method, the sampling scheme and the %N_dfa_ determination are given in S2 File.

#### Micro-topography of the topsoil in relation to water availability

Water availability for seed germination and seedling development was not measured directly as soil water content. Instead we characterised the micro-topographic variations of the topsoil layers; it is well known that micro-topography influences water availability [18], and that plants can benefit from higher water availability in pits and furrows [33]. As the relationship between micro-topography and water availability changes depending on meteorological conditions, we complemented topographic measurements with information regarding climatic and seasonal variations during the study, and we built a seasonal water availability index (see below).

Regarding micro-topography, variations were considered at the two different spatial scales. At the fine grained scale, we characterised the micro-topography using a rigid plane placed horizontally in alignment with the quadrats, with holes located precisely at the centre of each of the one hundred cells of the quadrat. The distance between the plane and the soil was measured with a graduated measuring stick for 36 of the 100 cells following a systematic grid. For non-measured cells, the distance between the plane and the soil was calculated as the mean of the neighbouring measured cells. For each quadrat, all distances were zero-centred to obtain the relative elevation of each cell within the quadrat. Then, this relative elevation was compared to the 8 neighbouring cells to create five classes of micro-topographic microsites: a) a 'pit': was the the lowest microsite (or in the penultimate position (rank 8, 8.5 (two *ex aequo* in a low position) or 9); b) 'low': rank 6, 6.5, 7 or 7.5; c) 'middle': rank 4, 4.5, 5 or 5.5; d) 'high': rank 2.5, 3 or 3.5) and e) 'top': rank 1, 1.5 or 2.

At the coarse grained scale, we used the standard deviation of the relative elevation of the microsites of one quadrat to characterise the heterogeneity of the topography.

Regarding climatic and seasonal variations during the study, we used data recorded in nearby sites. Daily rainfall and mean temperature were recorded in a meteorological station three kilometres away from the study site. We also built a Seasonal Water Availability Index (SWAI) using measurements of soil water content of the topsoil layer [0–15 cm] from CS615 probes (Campbell Scientific) in two sites close-by, under conditions similar to the experimental stand (i.e. young pine stands with low vegetation development and the same sandy soil texture). Data from the closest site (3 km) were only available for 2011, so we preferred to use data from a site farther away (30 km) after verifying that the daily soil water content measured at both sites in 2011 were highly correlated (r_pearson_ = 0.76, P<0.001) despite a period of missing data for this site. As it is difficult to transpose data from another site, even if it is close and very similar, we did not use these values of soil moisture to characterise the variation of soil moisture in our stand. Instead we built our SWAI by comparing the different intervals of time between each pair of surveys as follows:

- First we calculated a relative soil water content index as in Hartmann *et al*. [34]:

$$RSWC_{i}=\frac{SWC_{i}-SWC_{\text{min}}}{SWC_{\text{max}}-SWC_{\text{min}}}$$ (1)

Where: RSWC_i_ is the relative soil water content on day i; SWC_i_ is the water content measured on day i; SWC_min_ and SCW_max_ are the minimum and maximum values of soil water content measured. This index varied between 0 and 1; these were respectively the lowest and highest water content values measured during the course of the study.

- Second, we averaged this index for the interval of approximately 50 days between each pair of observations to create our SWAI index reflecting the seasonal water availability:

$$SWAI=\frac{\sum_{i=1}^{n\approx 50}RSWC_{i}}{n}$$ (2)

Values close to 0 of this SWAI indicate the most probable periods for water stress in the region. The seasonal increase of water availability is shown by an increase of this index. As biotic interactions are dependent on seasonal climatic variations, the calculation of this index is also relevant regarding the effect of vegetation surrounding gorse seedlings.

#### Neighbouring vegetation characterization

We characterised the development of neighbouring vegetation at the two spatial scales. Regarding the microsite scale, an index of vegetation development was recorded at each monitoring date within all quadrats. This index was recorded for all microsites in 2010, but only for microsites occupied by seedlings in 2011. The vegetation index was built as follows: a score was attributed to each of the 9 cells in the neighbourhood of the microsite (one central plus the 8 neighbouring cells) based on visual vegetation cover (0: 0–25%; 1: 26–75%; 2: 76–100%). The vegetation development index was calculated as the sum of the 9 scores which ranged from 0 to 18. In addition, for each year of the study, after the end of vegetative growth, aerial biomass of the vegetation was harvested from four areas of 20 cm^2^, adjacent to each corner of each quadrat. Then a single composite sample was dried and weighed per quadrat. Finally, at each observation date, and for each microsite, the vegetation index was refined following Eq 3.

$$VI_{ijt}=\frac{\sum COVER_{1-9t}\times Biom_{jt}}{\text{max}{\left(Biom\right)}_{t}}$$ (3)

Where, VI_ijt_ is the vegetation development index of microsite i, in quadrat j, at observation t;

Σ COVER_1-9t_ is the raw vegetation index at observation t; Biom_jt_ is the aerial biomass of quadrat j weighed in the year corresponding to observation t; max (Biom)_t_ is the maximum of aerial biomass weighed for the 72 quadrats in the corresponding year. Finally we grouped microsites based on three vegetation development classes of equal size (groups of microsites with low, medium and high development of vegetation), respecting the distribution of the tertiles of the vegetation development index.

The dry biomass harvested around each quadrat was used as an indicator of vegetation development at the coarse-grained scale.

### Data analysis

The response of a seedling depends largely on its date of emergence. Thus, we focused specifically on the response of different cohorts of seedlings (i.e. groups of seedlings whose presence was first recorded at the same census). With regard to seedling emergence, the effect of the sowing treatment was obvious. Sown and unsown areas were consequently analysed separately. Concerning seedling development, mortality and growth are two dependant processes. A low growth rate may indicate a plant that is not vigorous and healthy, which may lead ultimately to death. When the growth of a cohort of seedlings is analysed for a given lapse of time, some individuals present at the beginning of the interval are dead and absent by the end of the period. This 'not at random' loss of a part of the sample, preferential to seedlings with low growth rates, may lead to the spurious interpretation of statistical models [35]. Thus development of seedlings was analysed in two successive stages to answer the following questions: *1)* which individuals were dead and why? and *2)* among the survivors, what were the factors or variables that influenced growth (and finally the success of recruitment)?

#### The responses at the different scales

Emergence of new seedlings and mortality were first analysed at the microsite scales, and over the interval of time between two censuses (approx. 50 days). The microsite variables (classes of micro-topography and of vegetation development at this time) were used for these analyses as well as treatments applied to the stand (P fertilisation and sowing). Events that occurred in the interval between two censuses were also compared to the SWAI corresponding to the same lapse of time. As the responses at the microsite scale were measured repeatedly at different censuses, we also performed longitudinal analyses for the evolution of new seedling emergence, mortality and growth of seedlings during the course of the study in different kinds of microsites. Coarse-grained analyses (at the 1 m^2^ quadrat scale) were made by using variables characteristic to the quadrats (vegetation biomass harvested, heterogeneity of the micro-relief, and localisation in P fertilised or sown areas).

### Statistics

All analyses were performed with R software [36]. Selection of significant explanatory variables was made with a backward procedure, from saturated models with all explanatory variables and their interactions. The effect of the 'block' factor was always tested, and kept in the different models if significant. Pairwise multiple comparisons were made when we found that a multilevel factor significantly influenced the observed responses (package multcomp in R).

Dealing with binary responses (i.e. emergence of a new seedling in a microsite or death of a seedling), we used general linear modelling with logit link function (i.e. logistic regression) for analysis. We used general linear modelling with log link function for Poisson distributed count data (e.g. number of new seedlings per quadrat).

#### Fine-grained scale

Regarding the probability of seedling emergence between two censuses, we used mixed models with random intercepts to take into account the dependence of the microsites of the same quadrat (lme4 package in R). Concerning seedling mortality between two censuses, since we often had only one seedling per cohort and per quadrat, we could not fit mixed models with a random intercept for each quadrat (it is equivalent to allowing a random response for almost every seedling, and impedes the detection of the explanatory variable effects). Instead, we fitted conventional general linear models, and checked the significance of all model parameters using ordinary non parametric bootstraps with 2000 permutations (bootstrap package in R). Regarding longitudinal analysis, new seedlings emerged at each census. Thus we analysed the evolution of the proportion of different kinds of microsites without any seedling emergence during the course of the study with Cox proportional hazard models. The same kinds of models were used for survival analysis per cohort of seedlings in different kinds of microsites. We used mixed Cox models (library coxme in R) with a random intercept for each quadrat. As for growth, seedling size was normalised with logarithmic transformation. Then, we used mixed linear modelling with a continuous random effect for repeated measures of the size for the same individuals (library lme4 in R).

#### Coarse-grained scale

Analysis of deviance tables were made for general linear models regarding the number of emerged seedlings and recruits per quadrat on a yearly basis. The proportions of dead and recruited seedlings were analysed in the same way.

## Results

### General observations regarding seasonal variations of gorse regeneration

**Fig 1:**
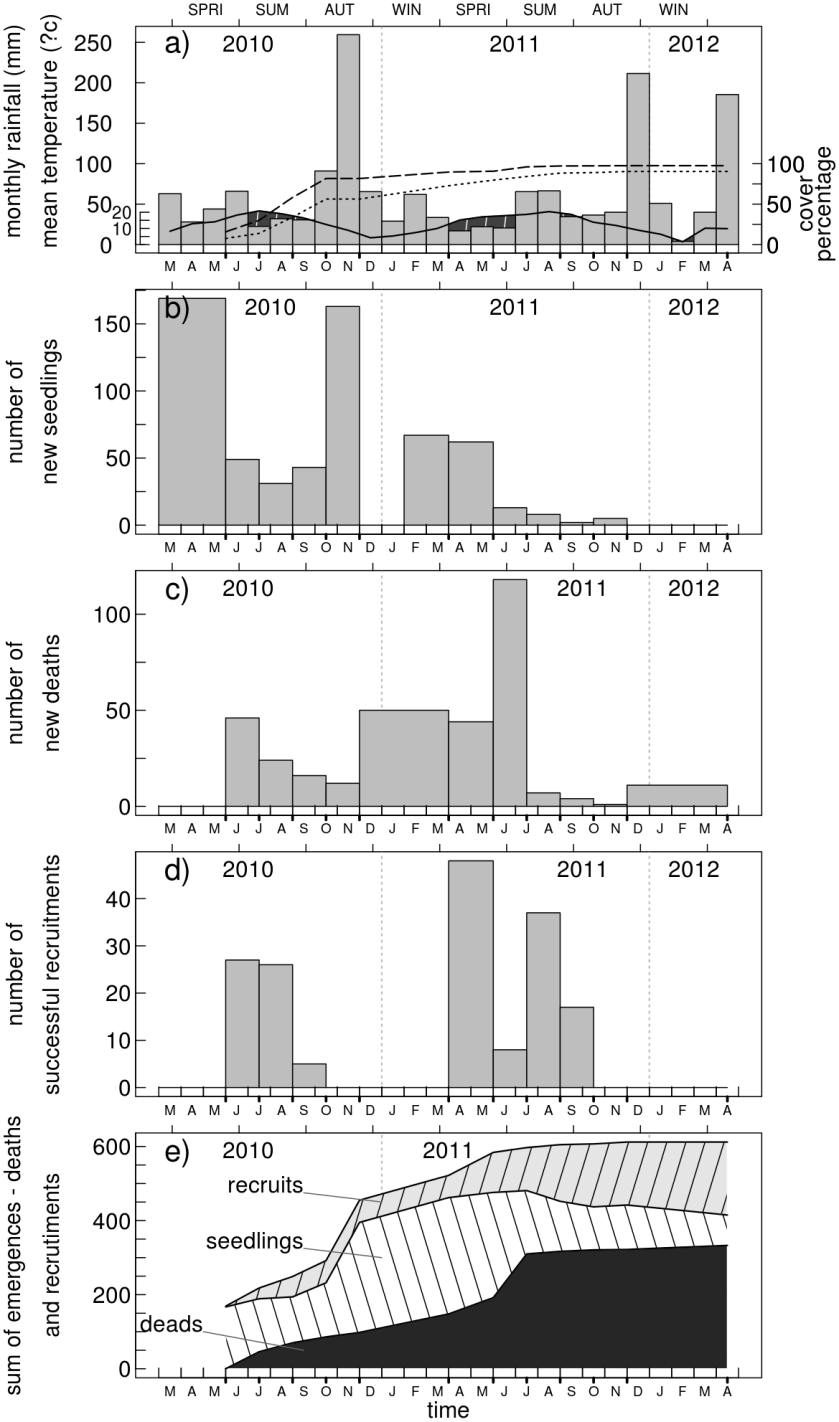
Number of different demographic events observed during the course of the study in relation to meteorological conditions and vegetation growth in the experimental stand. The 12 censuses made are indicated by a thick mark on the time axis. **a)** Monthly variation in temperature and rainfall (1 unit on the left y-axis equivalent to 1°C for the solid black temperature line, or 2 mm for rainfall histograms). The black shaded areas represent periods of water deficit risk. The evolution of the area of the stand covered by vegetation (right y-axis) is represented by dotted (no fertilisation) and dashed (fertilisation) lines. **b)** Number of new seedlings observed in the 72 quadrats (no seedling emergence occurred in December and January during the coldest period). **c)** Number of deaths. **d)** Number of new plants successfully recruited. **e)** The cumulated number of emergences is shown by the upper line. The cumulated number of deaths is shown by the lower black shaded area. The surviving plants are shared between the recruits (light grey area with diagonal lines-area) and the seedlings (white area with diagonal lines).

Important seasonal climatic variations occurred during our experiment (Fig 1a). The most noticeable meteorological events were: two periods with low rainfall and high temperatures suggesting water shortage (summer 2010 and spring-summer 2011), and high rainfall during both autumns and winters, with mean winter temperatures close to 0°C (Fig 1a). Colonisation of the overall stand area by vegetation was almost complete by the end of the study, and had already reached 80% in the fertilised area at the end of the first year (vs. 55% in the unfertilised area, Fig 1a). Seedling emergence was high in spring of both years (census n° 1 and 6), and there was also a large emergence peak in autumn 2010 (census n° 5), coinciding with a period of high rainfall and still relatively high temperatures (Fig 1a and 1b). Mortality was the highest in June in both years, and in particular in 2011, which coincided with three consecutive months of low rainfall (Fig 1a and 1c).

Mortality was also high in winter 2010–2011. We observed that the period immediately following emergence formed a critical stage for seedling survival. Indeed 151 of the 328 deaths (i.e. 46%) recorded during the survey were recorded following emergence. Periods of recruitment, when the plant size threshold was reached (S1 File), extended from spring until the end of summer in both years. A higher peak of recruitment success occurred in spring 2011, followed by a strong decline which was simultaneous with the third successive dry month (Fig 1a and 1d). A total of 612 gorse seedlings appeared during the study, 55% died and 32% were successfully recruited. The remaining 13% formed a seedling bank (e.g. [37]) with an uncertain future by the end of the study (Fig 1e).

Despite emergence in contrasting climatic conditions and within a more or less developed vegetation cover (Fig 1a and 1b), we found no significant difference in proportion of dead seedlings among the different seedling cohorts (Table 1). However, cohorts that appeared early had a high proportion of individuals that were recruited (Table 1). With the highest number of seedlings, and a high proportion that were recruited, the first cohort alone contributed to 46% of all seedlings successfully recruited by the end of the study.

**Table 1:** Size of the main cohorts of seedlings (cohorts n° 1 to 7) and their contribution to total recruitment, their respective proportion of deads, proportion of recruits, and non-recruited seedlings.

Cohort number	Number of seedlings	Proportion in all recruited seedlings	Number of deaths	Number of recruits	Number of living non-recruited seedlings	Proportion of deaths	Proportion of recruits	Proportion of living non-recruited seedlings
1	169	0,46	91	72	6	0,54a	0,42a	0,04a
2	49	0,13	21	21	7	0,43a	0,43ab	0,14ab
3	31	0,09	17	14	0	0,55a	0,45ab	0,00ab
4	43	0,04	26	7	10	0,61a	0,16bc	0,23b
5	163	0,14	107	22	34	0,66a	0,13c	0,21b
6	67	0,08	36	13	18	0,54a	0,19bc	0,27b
7	62	0,06	30	9	23	0,48a	0,15c	0,37b
Total	584	1,00	328	158	98	0,56	0,27	0,17

Different lowercase letters indicate significant differences in the proportions measured between cohorts (P<0.05).

### Influence of P fertilisation treatments

**Fig 2:**
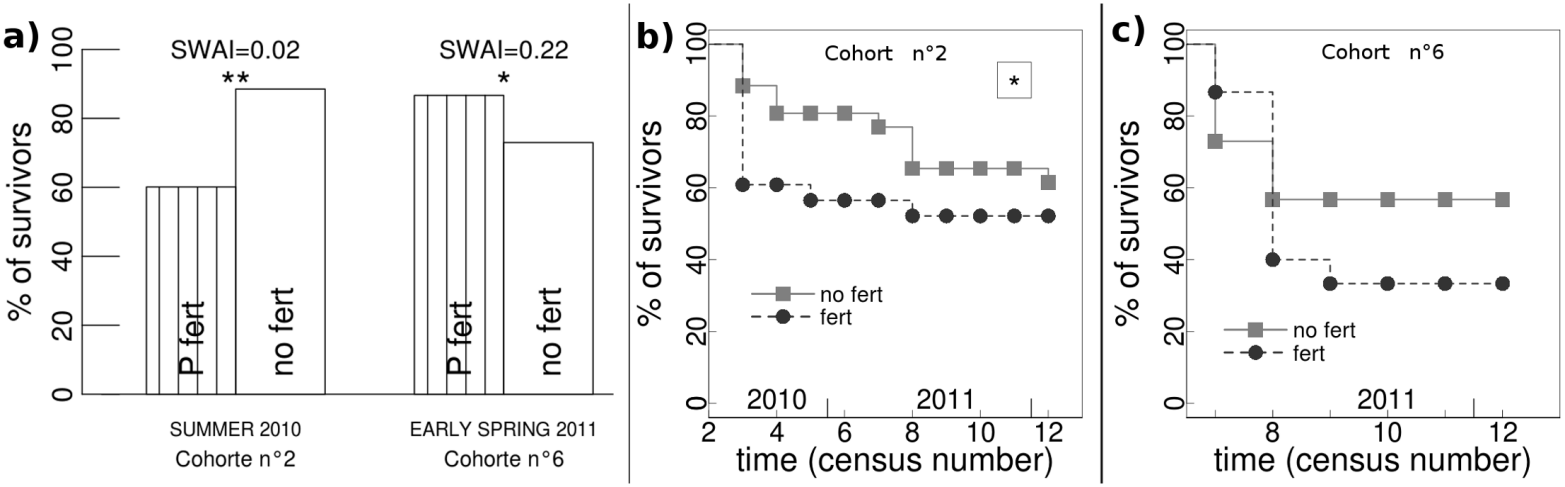
Effect of P fertilisation on seedlings survival. Significance of the P-fertilisation treatment is shown (**, P<0.01; *, P<0.05). **a)** Seedling mortality is shown for the interval of time following emergence at different seasons. The corresponding period and cohort number are indicated at the bottom of the figure. Seasonal Water Availability Index (SWAI) is shown on the top. **b)** and **c)** Results of mixed survival Cox modelling is shown for the same cohort of seedlings. Panels b and c correspond to the second and sixth cohort, as in panel a.

P fertilisation did not influence seedling emergence. Instead, its main effects were on seedling survival and seedling growth. During the dry summer 2010 (low SWAI), survival of seedlings that had just appeared was lower in fertilised areas, whereas in early spring 2011 (higher SWAI), their survival was lower in unfertilised areas (Fig 2a). The longitudinal analysis of seedling survival over time showed that: 1) this initial difference of survival with fertilisation can be maintained over time (e.g for the second cohort, Fig 2b); 2) shifting effects at different periods are also possible. For instance, for the sixth cohort, after higher mortality in the unfertilised area had been observed at emergence (Fig 2a), mortality was much higher in fertilised areas during the following census, in late spring 2011, during a period of low water availability (SWAI = 0,06). Thus the effect of fertilisation on the growth of this cohort was not significant when considering the whole course of the study (Fig 2c). At the coarse-grained scale, we found no effect of P fertilisation on seedling survival for either of the two years of study (Table 1b).

**Fig 3:**
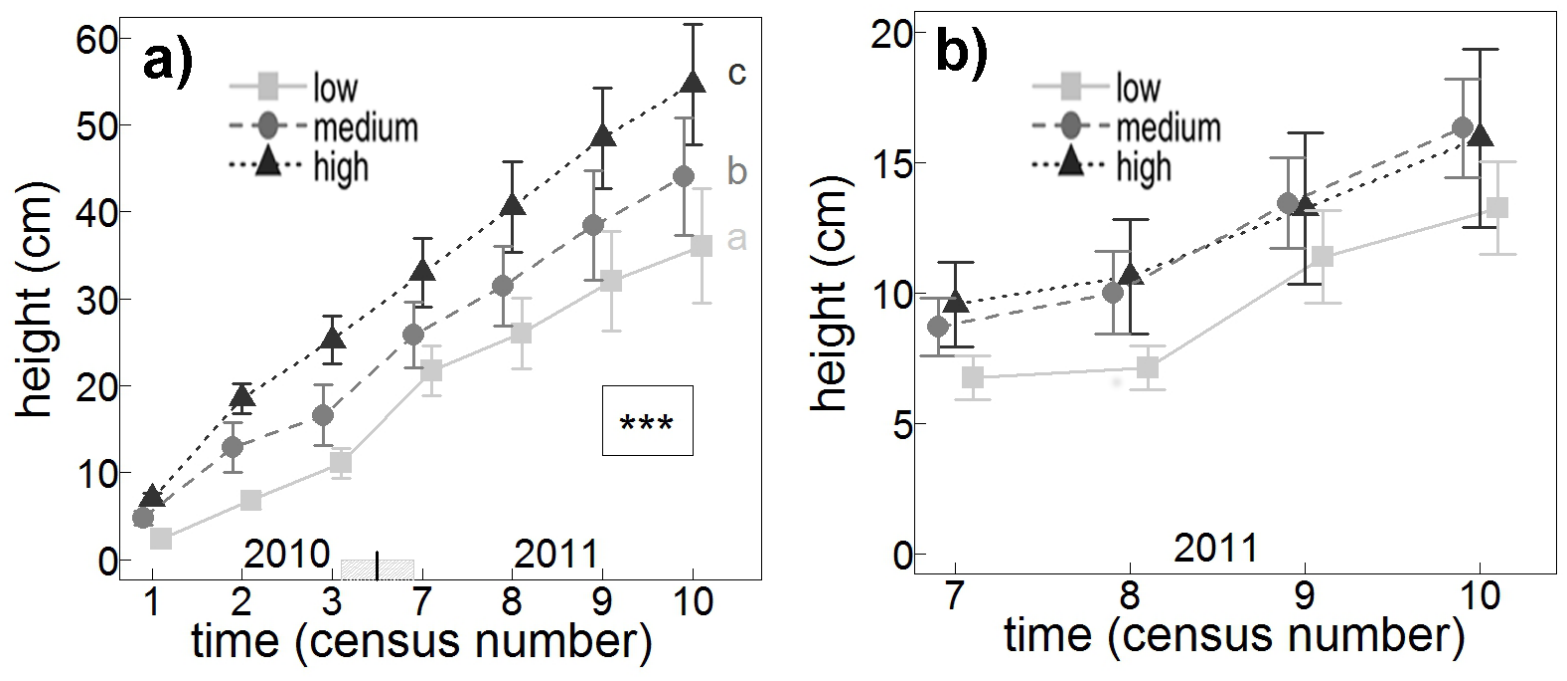
Seedling growth in relation to the development of the neighbouring vegetation in the microsite for the first cohort (a) and the fifth cohort (b), in fertilised plots. Seedlings were grouped into three classes of vegetation development based on the average values over all observations of the corresponding microsites. In a) the overall significance of the model is shown on the lower right-hand corner (***, P<0.001). Different lowercase letters indicate significant differences of height between different classes of seedlings (P<0.05). Almost no growth occurred from census 4 to 6 (autumn-winter time) and the corresponding period was withdrawn from analysis to linearise the relationship of growth with time (see the shaded area on the time axis). Note the different scales on the y-axis in a) and b).

Regarding growth, at the fine-grained scale, for seedlings that survived until the end of the study, P fertilisation was the main explanatory variable that enhanced growth, indicating that the size threshold for successful recruitment was reached faster in fertilised plots (result not shown). In fertilised areas, for early emergent seedlings (first cohort), the observed growth was higher where vegetation development was also higher over the whole study (Fig 3a). For most seedlings that emerged later in fertilised areas, growth was not correlated with vegetation development (Fig 3b). For all seedlings (i.e. early or late) that appeared in unfertilised areas, there was no difference of seedling growth with regard to vegetation development (result not shown). Considering the coarse-grained scale, for seedlings that appeared in 2010, a higher number of plants were successfully recruited in P fertilised areas in the same year (Table 2a). This confirmed the positive effect of fertilisation on growth at the larger scale. Moreover, we found a higher proportion of seedlings recruited in P fertilised areas with high vegetation development (Table 2b). This is consistent with Fig 3a regarding the first cohort, because 68% of the recruited individuals came from this cohort. For individuals that were successfully recruited one year later in 2011, their proportion per quadrat increased in fertilised areas (Table 2b). For seedlings that appeared in 2011, we found no effect of P fertilisation on growth.

**Table 2:** Results regarding emergence, death and successful recruitment at the coarse-grained scale.

**a)**	Year considered	2010		2011	
Number of emergences	Explanatory variables / Sowing treatment	Sown	Unsown	Sown	Unsown
	Heterogeneity of micro-topography^a^	0,02 (+)	-	-	-
	Vegetation development	-	-	-	-
	Fertilisation	-	-	-	-
Number of recruits	Year considered	2010		2011	
	Explanatory variables / Sowing treatment	Sown	Unsown	Sown	Unsown
	Heterogeneity of micro-topography	0,01 (+)	-	-	-
	Vegetation development	-	-	-	-
	Fertilisation	0,02 (+)	-	-	-
	Fertilisation x vegetation development^b^	-	-	-	-
**b)**	Year of recruitment	2010		2011	
Proportion of deads	Explanatory variables / period of observation	in 2010	in 2011		
	Heterogeneity of micro-topography	-	-	-	
	Vegetation development	-	-	-	
	Fertilisation	-	-	-	
	Fertilisation x vegetation development	-	-	-	
Proportion of recruits	Year considered	2010		2011	
	Explanatory variables / period of observation	in 2010	in 2011		
	Heterogeneity of micro-topography	-	-	-	
	Vegetation development	-	-	-	
	Fertilisation	-	<10^-2^ (+)	-	
	Fertilisation x vegetation development	0,04 (+)	-	-	

In a) the numbers of new seedlings and recruits per quadrat are shown. In b) the proportions of deaths and recruits per quadrat are shown.

^a^: based on the standard deviation of cell elevation within the quadrats.

^b^: interaction between fertilisation treatment and vegetation development.

A dash indicates a non-significant effect of the explanatory variable, otherwise the level of significance is indicated, as well as the direction of the effect ((+) indicates that the increase of the explanatory variable, or the application of the treatment led to an increase of the dependant variable).

Regarding P fertilisation and symbiotic nitrogen fixation, we observed a mean increase of 20% N_2_ fixation rate (%N_dfa_) in the gorse seedlings in fertilised areas in 2010. Conversely, the fixation rate was 10% lower in fertilised than in control areas (Table 3). However, these intra-annual differences were only marginally significant (P<0.1).

**Table 3:** Variations of the rate of symbiotic nitrogen fixation (%N_dfa_) with the year of study and P fertilisation each year.

Year (***)		Fertilisation treatment (n.s)				
	Mean ± SE	n		Mean ± SE		n
2010	54.5 ± 6.2	22	Fertisation	62.8 ± 8.4	b	12
			Control	41.9 ± 7.4	a	10
2011	77.3 ± 2.8	65	Fertisation	72.7 ± 4.0	a	35
			Control	82.6 ± 3.8	b	30

Information given in brackets indicates the result of the ANOVA in the whole sample (***, P<0,001). Different lowercase letters indicate only marginal differences (P<0.1) between classes performing within-year tests.

### Influence of micro-topography with regard to seasonal water availability (SWAI index)

**Fig 4:**
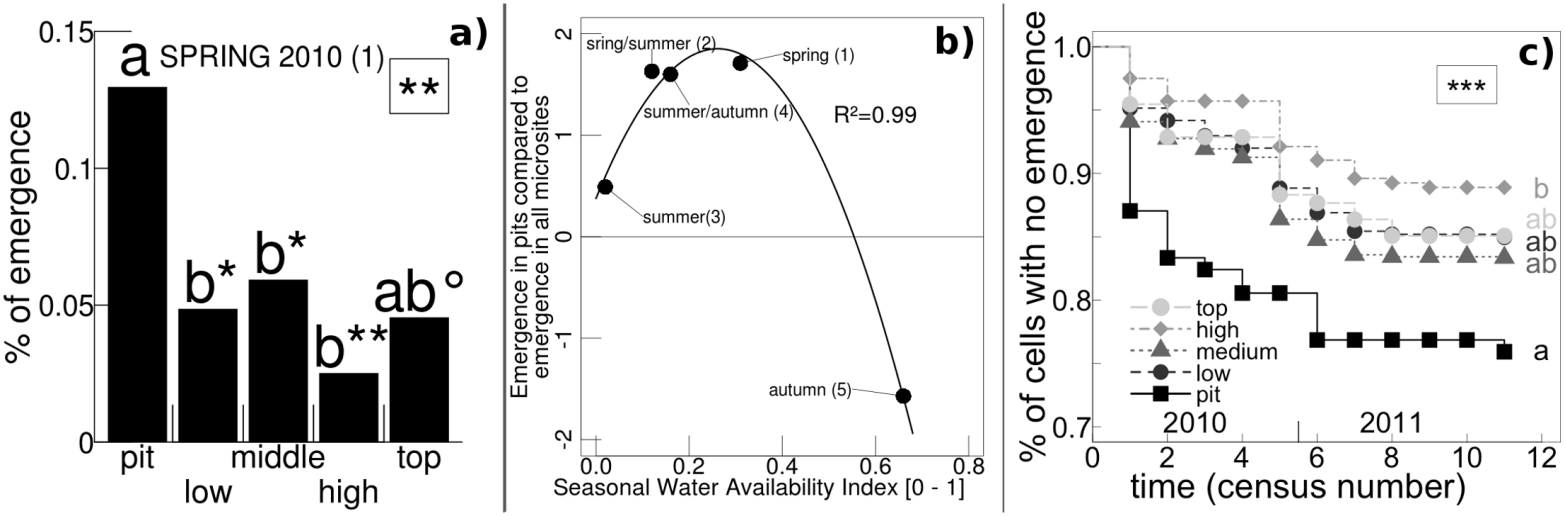
Relationship between micro-topography and frequency of emergence in sown areas. a) The proportion of microsites where emergence occurred in spring 2010 (first census) is shown relative to micro-topographic classes. The overall significance of the mixed logistic regression is shown in the upper right-hand corner (**, P<0.01). Different lowercase letters indicate difference between micro-topographic classes (P<0.05). Symbols next to these letters indicate the significance of the difference with the pit class (**, P<0.01; *, P<0.05; °, P<0.1). b) For each of the 5 censuses made in 2010 (see number in brackets), the standardised difference between the proportion of micro-pits where emergence occurred and the mean proportion of emergence for all microsites is regressed as a function of the Seasonal Water Availability Index. Values above and below zero indicate higher and lower proportion of emergence in micro-pits, respectively. The line shows the adjustment by a polynomial function of order 2. c) The evolution of the proportion of microsites with no emergence during the course of the study is shown relative to micro-topographic classes. The overall significance of the mixed Cox proportional hazard model is shown in the upper right-hand corner (***, P<0.001). Different lowercase letters indicate difference between micro-topographic classes (P<0.05).

The main effect of micro-topography was on seedling emergence, and was detected only in areas sown with gorse seeds. Under these conditions, in 2010, at the fine-grained scale, wide variations of emergence frequencies between topographic classes of microsites were observed, mainly in micro-pits (Figure A in S3 File). At two observation dates (in spring and summer-autumn), emergence frequencies between micro-topographic positions showed significant differences (Figure A, panels a and d,in S3 File). For the first cohort (i.e in spring), pairwise multiple comparisons showed higher emergence in micro-pits compared to other micro-topographic positions (Fig 4a). In general, emergence in micro-pits was higher during periods of moderate rainfall and low to intermediate water availability (Fig 4b). In contrast, during periods of very low rainfall and SWAI (summer, Figure A, panel c, in S3 File), emergence frequencies in pits did not differ. In the case of abundant rainfall and very high water availability (autumn), emergence tended to be lower in this position (Figure A, panel e, in S3 File). This resulted in a unimodal curve for the relationship between the differences of emergence in pits compared to emergence in the whole sample and the SWAI (Fig 4b). In 2011, we found no effect of micro-topography on seedling emergence at the different census times (Figure B in S3 File). Over the course of the study, emergence in micro-pits was the highest, but only differed significantly from the ‘high’ micro-topographic class (Fig 4c), due to the large difference between these micro-topographic classes observed in spring for the first cohort (Fig 4a).

At the coarse-grained scale, quadrats with heterogeneous micro-topography showed a higher number of new seedlings in sown areas in 2010, but not in 2011 (Table 2a). Regarding the number of seedlings recruited per sown quadrat, we found an increase related to the heterogeneity of the micro-topography (Table 2a). This was due to the higher emergence of the seedlings mentioned above, and to the correlation between the number of seedlings that emerged and the number that were successfully recruited in all quadrats (result not shown).

### Other effects regarding surrounding vegetation in relation to seasonal water availability

**Fig 5:**
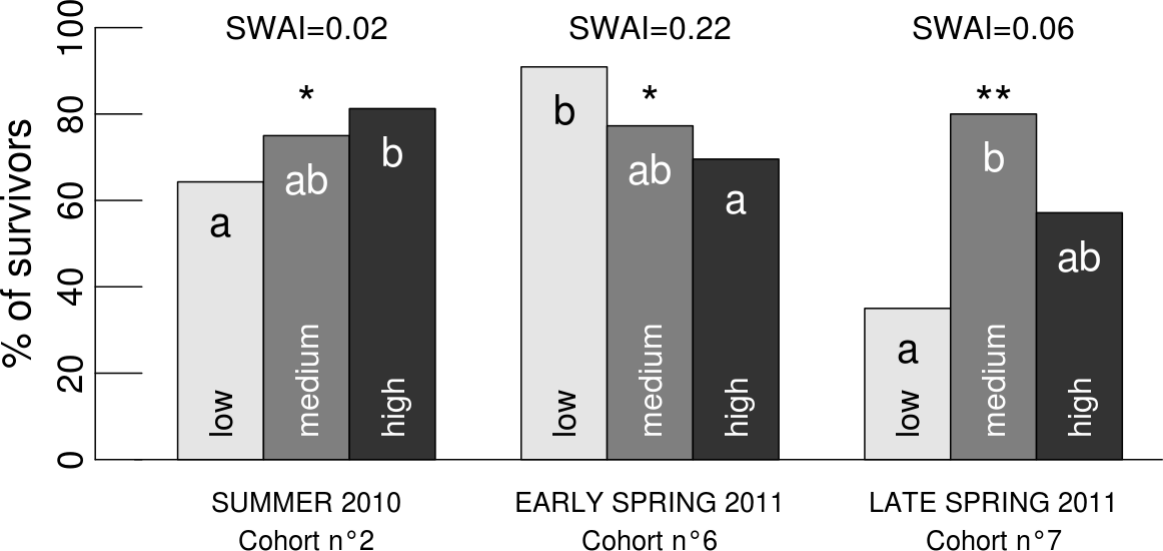
The effects of neighbouring vegetation around microsites on seedling survival following emergence. Significance of the effect of vegetation development classes is shown (**, P<0.01; *, P<0.05). These classes of vegetation development are based on the seedling vegetation development index at emergence. Lowercase letters indicate a significant difference between classes (P<0.05). Seedling survival is shown for the interval of time following emergence at different seasons. The corresponding period and cohort number are shown on the x axis. The corresponding Seasonal Water Availability Indices (SWAI) are shown at the top.

We found contrasting effects of surrounding vegetation development on seedling mortality at the fine-grained scale, depending on the level of seasonal water availability. In summer 2010 and late spring 2011 (i.e. low SWAI), mortality of seedlings that had just appeared was lower when vegetation development was high (Fig 5, cohort n°2 and 7). We observed that during these intervals, seedlings that survived did not grow higher in these micro-sites with higher vegetation development (result not shown). In early spring 2011 (higher SWAI), the opposite effect occurred (Fig 5, cohort n° 6). It is noticeable that, at the coarse-grained scale, the effect of vegetation development was not detectable when considering all seedlings that emerged in 2010 and 2011 (Table 2b).

## Discussion

### The role of P availability for gorse regeneration

The most obvious effect of P fertilisation treatments on gorse regeneration was an improvement of seedling growth, which led ultimately to a higher number of successfully recruited seedlings in fertilised areas. This is consistent with other studies which found that P-availability limits legume growth [9]. Moreover P is the main limiting nutrient in the region [6]. Conversely, in unfertilised areas, seedling development may have not changed because of this major limitation.

In P fertilised areas, we found an even higher growth for early emergent seedlings in microsites with higher vegetation development (Fig 3a). This result cannot be explained because of etiolation of gorse seedlings due to shading. Indeed, the mean height of the neighbouring vegetation layer in spring 2011 was only 13 cm (S1 File), and internode elongation of gorse seedlings in the shade is limited [27]. Instead we propose that in the case of early emergence after stand clear cutting, interspecific competition for the resources was low. Early emergence is known to benefit seedling growth [38,39]. In our study, thanks to the early establishment of their root and shoot systems, gorse seedlings may have been less sensitive to the interspecific competition that increased later. Once the phosphorus limitation was removed, the development of seedlings might have been optimal where local fertility was the highest (i.e. in patches with a high vegetation development index), which could explain our results. Conversely, in case of late emergence, when the seedlings appeared the neighbouring vegetation was already well developed. Thus in the presence of high vegetation development, high interspecific competition may have balanced the positive effect of increased nutrient availability (Fig 3b).

In addition, in 2010 we found a marginally significant positive effect of fertilisation on the rate of biological nitrogen fixation (%N_dfa._, Table 3). Fertilisation may have favoured the establishment of symbiosis for young seedlings. In 2011, the slight decrease of the fixation rate in fertilised areas is counter-intuitive but, as in 2010, this difference was weak and only marginally significant. Such a pattern in 2011 might be the consequence of a small sample size during a period of variable and increasing fixation rate from the seedling stage to the adult stage. Indeed, in an additional sample made in 2013, there was no difference of fixation rate between fertilised plants and control plants (unpublished data) and this later result is consistent with the low limitation of nitrogen fixation rate by phosphorus availability found for the mature legumes of this forest [40] as well as in most regions of the world [9]. Overall P fertilisation did not have much influence on the functioning of the symbiosis (see also S2 File).

Results in relation to seedling survival in response to P fertilisation are better explained by taking into consideration the importance of water availability. Indeed, plants in fertilised areas have less developed and/or more superficial root systems which are concentrated in soil layers of high nutrient availability (e.g. [41]). Consequently, seedlings in fertilised areas may be more sensitive to drought that begins with a decrease in soil moisture in the topsoil layer. This probably explains the decreased survival in fertilised areas during dry periods (Fig 2a, cohort n°2). Conversely, during moister periods, seedlings benefited from higher P availability (Fig 2a, cohort n°6). Given this contrasting effect regarding seedling survival, no effect of P fertilisation can be detected when considering longer time lapses (Table 2).

### The role of water availability for gorse regeneration

Water availability was not measured directly in our experimental stand. Thus our interpretations regarding water availability presented here have to be considered with care. On the other hand, it is important to note that variations of our seasonal water availability index (SWAI) were totally consistent with the meteorological variations observed during the course of the study, which suggests that the use of our index is fairly justified.

The general observation of seasonal variations of gorse regeneration (Fig 1) highlights the importance of seasonal rainfall and thus of water availability. Most of the seedlings emerged in autumn 2010 when rainfall was high, and most of the seedlings died in spring-summer 2011 during an important dry period. Results regarding microsite characteristics (micro-topography and neighbouring vegetation development) provided additional evidence of the importance of water availability.

The variation of emergence frequency in micro-pits with seasonal water availability suggests the specific role of local soil moisture around seeds. The increase in local soil moisture of micro-topographic pits is well described in farming systems where ridges and furrows are created during soil preparation [33,42]. Thus, in the case of moderately low water availability (spring, spring / summer and summer / autumn censuses in Fig 4b), the topsoil layer in these pits may have had higher water contents, which could have favoured germination of gorse seeds and resulted in the higher emergence frequencies observed under those conditions. Conversely, in the case of more severe drought (summer census), the whole topsoil layer may have been equivalently dry and micro-pits could have lost their germination advantage. In the case of abundant rainfall (autumn census), a negative effect due to an excess of water in pits could be a possible explanation for the lower emergence in autumn in this topographic class. Consequently, we found a unimodal relationship along the whole gradient of water availability (Fig 4b). As the first cohort contained the highest number of seedlings and appeared in conditions where emergence increased in micro-pits, the effect linked to micro-topography remains detectable at the coarse-grained scale (Table 2a). The importance of micro-topography on *Ulex parviflorus* seedling emergence has also been shown [43]. In that study, emergence was improved in high positions which is contrary to our results. However, the characterisation of the micro-topography was different, and was composed of only two classes (high and slopes) without a class dedicated to micro-pits.

The decrease in mortality just after emergence in the presence of important vegetation development during dry episodes (Fig 4, cohorte 2 and 7) suggests that soil moisture is an important factor controlling seedling survival as well. When the seedlings emerged during dry periods, the presence of high vegetation cover may have limited evaporation thus providing higher soil moisture, as shown in many studies [13,44]. Such a facilitating effect could widen the physical space where seedlings can survive towards more stressful habitats [45]. Conversely, during wetter periods (Fig 4, cohort 6), greater vegetation development around gorse seedlings may have increased competition for light and nutrients. At the coarse-grained scale, no effect linked with neighbouring vegetation was detectable (Table 2).

### Gorse regeneration niche and regeneration habitat

The strategy of regeneration that appeared in our results—the importance of the early emergence after a disturbance, sensitivity to water availability and the importance of the facilitation by nurse plants during dry episodes—is totally consistent with former results regarding the Mediterranean gorse, *Ulex parviflorus* [15,39]. As to the gorse regeneration niche, we showed evidence that P and water availability are important ecological factors for species regeneration. High water and nutrient stresses exist in our system due to the presence of nutrient-poor sandy soils. Thus our results are consistent with previous work which showed that niche-based processes play a decisive role in stressful communities [46].

Moreover, many significant and contrasting effects were detectable during the fine-grained analysis, whereas only two results emerged at the coarse-grained scale: 1) the number of new seedlings increased with micro-topographic heterogeneity in the quadrats, certainly due to the higher frequency of micro-pits; and 2) recruitment was enhanced in fertilised areas, related to the improvement of seedling growth. No explanatory variable could be identified at the coarse scale regarding seedling mortality. In the context of our study, habitats with marked micro-topography in fertilised areas appeared more favourable for the recruitment of the species. In these habitats, appropriate soil moisture for germination may be found more frequently in micro-pits, and phosphorus availability for seedlings development was certainly higher there. Yet the generalisation and assignation of these habitats to the species regeneration niche remains questionable for two reasons.

Firstly, it is important to take the high variability observed during the fine-grained analysis into consideration. P and water availability depended on the interaction between microsite characteristics and stochastic climatic variations. For instance, the increase in phosphorus availability removed a major limitation on growth but, on another hand, survival decreased in fertilised areas for some cohorts during dry periods. Thus, the effect of fertilisation on recruitment of new plants was contrasted, as reported in former studies (e.g. [47]). This climatic and stochastic influence challenges the relative strength of the different effects observed during this study in the long term, and from our results, a generalisation regarding the habitats most suitable for regeneration is difficult.

Secondly, the role of micro-topography that was observed at the coarse-grained scale is totally consistent with results found for the first cohort of seedlings, which accounts for 46% of recruited individuals. The difference in proportion of micro-pits where new seedlings appeared, compared with the rest of the micro-topographic classes at the end of the study, is almost totally determined by the initial difference that occurred when the first cohort appeared (Fig 2c). Thus, this single cohort is likely to have influenced the coarse-grained scale results. Even if gorse is considered as a pioneer species, it can also recruit, grow and reproduce later in the succession [23] like other *Ulex* species in the Mediterranean region [48]. Results influenced by only one cohort that appeared early after a clear-cut cannot be generalised to represent the whole regeneration process of the species in the long term.

### Implications for the regeneration niche concept

In his early definition, Grubb stated that the regeneration niche ‘includes elements of both address and profession’ and is partly composed of the 'habitat niche' or 'the physical and chemical limits tolerated by the mature plant in nature'[1]. One might find Grubb’s definition puzzling considering the ambiguity existing between niche and habitat. Following Hutchinson [49], the niche of a species has largely been accepted as being defined in virtual ecological space by the n-dimensional hypervolume where environmental conditions are suitable for the species to maintain a reproductive rate higher than or equal to 1 [50]. In contrast, the habitat of a species has a strong spatial extension in the physical world or geographical space [51]. The concept of the niche suffers from a high diversity of meanings and uses [51,52]. While we recognize that this problem probably obscures the current debate regarding regeneration niches, our results also call for clarification of Grubb’s concept and definition.

In field studies, probably influenced by the ‘habitat niche’ definition of Grubb, the characterisation of regeneration niches has frequently been made on the basis of distinct habitats or micro-habitats distributed along a transect between the centre and the periphery of a gap, along a topographic gradient, or on different substrates (e.g. [19,20,53–55]). However, high variability is often observed within each habitat, as in the present study. Indeed, the recruitment of woody species is known to be highly variable in space and time [30]. Finally, the ecological determinism of this variability remains poorly understood and the importance of the regeneration niche concept has no definite answer.

Instead, analysing recruitment patterns through the duality proposed by Hutchinson [49,50], taking into account the correspondence between the niche of the seedlings (in ecological space) and the physical world where the seedlings appear and develop (the ‘biotope’ sensu Hutchinson), may provide a powerful way to highlight the ecological determinism of recruitment that can be hidden by the wide variability observed in the field. To do so, determining the appropriate scale (both spatial and temporal) of the study is crucial, as in any attempt to highlight specific ecological processes [56].

## Conclusion

This *in situ* study showed that P availability controls gorse seedling growth and the time necessary to reach young shrub size. Water availability appeared to impact the germination and seedling survival of the species. However, effects of phosphorus and water availabilities depended on the interaction between microsite characteristics and climatic variations. Consequently, in the same kind of microsites, contrasting effects appeared over time during the succession of favourable and unfavourable periods, and between the different stages of the recruitment process. All in all, we found evidence that P and water availability are important ecological factors shaping the regeneration niche of the species, but we found weak evidence that any habitat or micro-habitats could be continuously appropriate for the regeneration of the species in the long term. These results challenge the early definition of the regeneration niche proposed by Grubb. Future studies regarding regeneration niches should take into account to a greater extent, the correspondence between the niche of the seedlings (in the ecological space) and the physical world where the seedlings appear and develop.

## Supporting Information

S1 File Determination of the size threshold for the end of the regenerative phase. (DOCX)

S2 File Rate of symbiotic nitrogen fixation (%N_dfa_) and N and P contents of gorse seedlings in response to P fertilisation. (DOCX)

S3 File Supplementary results regarding the frequencies of seedling emergence and the different micro-topographic positions. (DOCX)
