# Supplementary material for: Weak Evidence of Regeneration Habitat but Strong Evidence of Regeneration Niche for a Leguminous Shrub
Source: PLoS One. 2015 Jun 22;10(6):e0130886. doi: 10.1371/journal.pone.0130886 (PMC4476804; doi:10.1371/journal.pone.0130886)
Supplement: S1 File — (DOCX) [file pone.0130886.s001.docx]

**S1 File.** Determination of the size threshold for the end of the regenerative phase.

*Justification*

Seedlings have, by definition, small shoots and toots. In comparison with mature plants, their ability to capture resources and their nutritive and energetic reserves are limited. Consequently, many woody seedlings have high mortality and low growth rates [1]. When access to resources increases, due to an increase of shoot and root size, and particularly with better light access, growth rate increases too [1]. As a consequence, young woody plants show a phase of exponential growth [e.g. 2]. Three criteria were used to characterise successful recruitment: *1)* a size threshold from which the number of death observed was almost zero, *2)* a size threshold from which growth rate of gorse plants increased sharply, and *3)* the measurement of the mean height of the herbaceous layer in the stand, as an indicator of the height that gorse plants need to reach to get a better access to light.

*Method*

*1)* D*ecrease in mortality:* the distribution of death frequency was observed as a function of the last plant size recorded before death (Figure A, panel a).

*2)* G*rowth rate increase:* the growth of young gorse plants during the interval from March to May 2011 was analysed as a function of their initial size (Figure A, panel b). This interval was chosen because: *i)* in 2010, not enough individuals had reached the mature phase to be able to observe a difference in growth rate from a size threshold, and *ii)* in 2011, drought strongly disrupted the growth of gorse plants that occurred after May.

*3)* *Mean height of the vegetation layer:* this measurement was made in summer 2011, outside the observation quadrats, using a horizontal framework placed in alignment with 30 new emergent seedlings (half in fertilised areas). This framework included 25 holes, at the intersections of a square grid with a mesh size of 5 cm. For each hole, the height of the vegetation was measured at the contact between the stick and the vegetation with a graduated stick.

*The selected size threshold*

The mortality observed during the study was strongly dependent on the size of the seedlings. Only 1.6 % of deaths were observed after seedlings had reached a size of 12 cm (Fig S1a). Between March and May 2011, below a size of 11.6 cm at the beginning of the interval, growth was very low. Above this size, growth increased significantly with the initial size (Fig. S1b). Finally, the mean height of the vegetation measured in 2011 was 13.5 cm, with no significant difference between fertilised areas and control (there was a difference in cover, but not in height). This indicates that close to this height, access to light was likely to be improved.

Based on these three consistent results, a size of 12 cm was selected to identify plants that were successfully recruited.


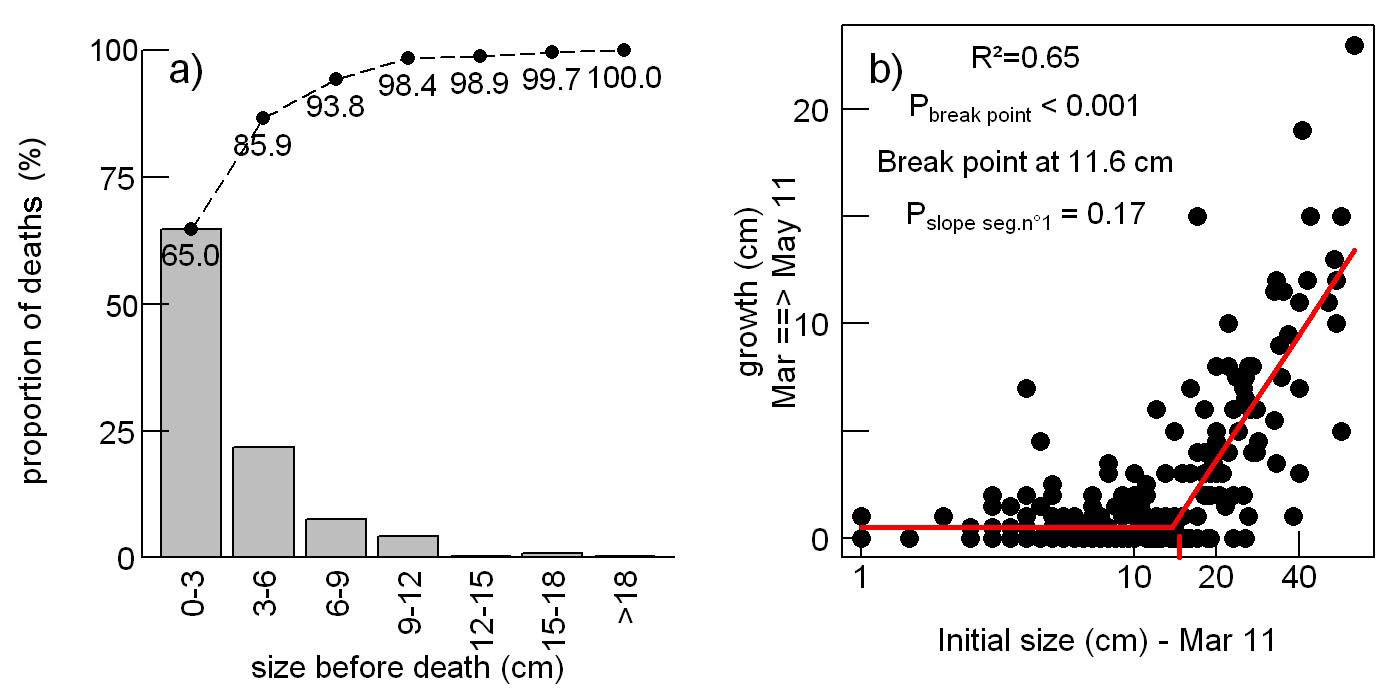


**Figure A.** Size threshold for successful recruitment. a) The accumulated percentage of death is indicated by the dotted line based on the last size recorded before death. N= 328. b) The initial size of plants is shown with a logarithmic scale on the x-axis for ease of visualisation (many points between 5 and 15 cm), but results remain strictly unchanged using a natural scale. Break point is highly significant (*P*<0.001), and its value, based upon the segmented regression in the natural scale, is indicated. Red lines correspond to the two segmented regression relationships. Slope of the first segment is not significantly different from 0

**References**

1. Closset-Kopp D, Chabrerie O, Valentin B, Delachapelle H, Decocq G (2007) When Oskar meets Alice: Does a lack of trade-off in r/K-strategies make Prunus serotina a successful invader of European forests? For Ecol Manag 247: 120–130. doi:10.1016/j.foreco.2007.04.023.

2. Tremblay MJ, Rossi S, Morin H (2011) Growth dynamics of black spruce in stands located between the 51st and 52nd parallels in the boreal forest of Quebec, Canada. Can J For Res-Rev Can Rech For 41: 1769–1778. doi:10.1139/x11-094.
