# Supplementary material for: Weak Evidence of Regeneration Habitat but Strong Evidence of Regeneration Niche for a Leguminous Shrub
Source: PLoS One. 2015 Jun 22;10(6):e0130886. doi: 10.1371/journal.pone.0130886 (PMC4476804; doi:10.1371/journal.pone.0130886)
Supplement: S2 File — (DOCX) [file pone.0130886.s002.docx]

**S2 File .** Rate of symbiotic nitrogen fixation (%N_dfa_) and N and P contents of gorse seedlings in response to P fertilisation.

*Justification and sampling protocol*

According to McLaughlin *et al.* [1], if a deficiency in phosphorus limits the proportion of nitrogen derived from atmosphere (%N_dfa_) fixed by a leguminous plant, it should result in lower N and P contents in the plant tissues. Thus, to measure the impact of phosphorus fertilisation on symbiotic nitrogen fixation by gorse seedlings, each year we measured the N and P contents, as well as the %N_dfa,_ in an additional sample of seedlings (aerial parts), that were located outside the quadrats of observation. %N_dfa_ determination was done based on the ^15^N isotopic dilution method [2] (see below).

At the end of the first season of vegetation growth (i.e. Automn 2010), three size classes of gorse seedlings were determined from the distribution of the tertiles of the length of all seedlings in the observation quadrats ('C1': 0-6'cm; 'C2': 6.1-16 cm, 'C3': > 16 cm). Then, three samples of each size class were harvested respectively in fertilised and control areas, in each block. In total, 54 gorse plants were sampled (3 blocks x 2 fertilisation treatments x 3 sizes of plant x 3 replicates). These gorse plants were sampled together with an equivalent number of non leguminous plants used as a reference (see below). In 2011, the same protocol was followed, except that we added a fourth class of plant size to take into account the possible evolution of the %N_dfa_ for plants that had grown higher ('C4': > 47 cm, percentile 90 at the autumn 2011), i.e. a total of 72 samples.

*The Isotopic dilution method*

When a comparison is made between a legume, and a neighbouring plant that does not fix atmospheric nitrogen, and whose rhizospheres are located in the same soil volume, the difference between their isotopic ratios [^15^N]/[^14^N] is an indicator of the proportion of nitrogen derived from the atmosphere in the tissues of the legume [3]. Calculations to determine the %N_dfa_ are indicated below in equations 1 and 2. They require the intermediate calculation of the ^15^N enrichment (δ^15^N, equation 1) of legumes and reference plants relative to the atmosphere, as well as the ^15^N enrichment of a legume for which 100 % of the nitrogen comes from atmosphere (δ^15^N_fix_):

δ^15^N_plant_ = (([^15^N]/[^14^N]_plant_ - [^15^N]/[^14^N]_atm_) / [^15^N]/[^14^N]_atm_ ) x 1000 (1)

%N_dfa_ = [(δ^15^N_ref_ - δ^15^N_leg_) / ( δ^15^N_ref_ - δ^15^N_fix_)] x 100 (2)

Where: %N_dfa_: nitrogen fixation rate of the legume, δ^15^N_ref_: δ^15^N of the non leguminous reference plant used, and δ^15^N_leg_: δ^15^N of the legume. The δ^15^N_fix_ of gorse is -0,55 ‰ [4]. This isotopic method is considered to give reliable results when there is large difference between δ^15^N_leg_ and δ^15^N_ref_ [5]. To ensure that this difference was large enough, the whole surface of the stand was labelled at the end of the winter following the clear cut and soil preparation (i.e before vegetation started to grow) by homogeneous spraying of ^15^NH_4_Cl corresponding to a non fertilising dose of 0.07 kg ^15^N ha^-^.

*Choice of the non leguminous reference plants*

It has been shown that *Erica scoparia* and *Calluna vulagris* are appropriate to serve as non legume plant for the use of the method, and that the δ^15^N is constant in the different tissues of gorse plants [6]. Therefore, for each sample, we harvested aerial tissues of the gorse plant, and a non-leguminous plant which had a similar size and was no farer away than 0.5 m. *E. scoparia* and *C. vulagris* were chosen preferentially, but they were scarce in the stand whose vegetation was mainly herbaceous. Therefore, we had to reduce the number of replicates in a few plots.

*Measurements of the* δ^15^N*, N and P contents.*

The samples were dried, weighed and ground in a ball mill (Retsch PM4 planetary grinder, Retsch, Haan, Germany) before N content and δ^15^N determination by spectrometry (“sector field” ICP mass spectrophotometer). Their P contents were determined by mineralisation with nitric acid [7]. When the dry weight of plant material was inferior to 300 mg (small samples, for the smallest seedlings) and P content could not be determined individually, the samples were pooled according to size class and fertilisation level.

*Correction of the %N_dfa_*

As far as plants of small size are concerned, the initial stock of nitrogen in the seed is an important part of their nitrogen mineral mass (e.g. 37.7% and 10.9 % for individuals of the first two size classes in our sample, based on the determination of nitrogen mineral mass in 10 seeds). Therefore, their isotopic ratio is influenced by this initial stock of nitrogen, and a correction of the %N_dfa_ was made for the classes C1 and C2. Gorse plants maintain high fixation rates, even when the N contents of soils increase [8]. Moreover, measurements of the %N_dfa_ of mature gorse plants in the region were always high (between 60 and 100% [4]). Therefore, we assumed that the isotopic ratio of gorse seeds was close to that of a gorse plant with a %N_dfa_ of 100% (i.e. δ^15^N_seed_ ≈ δ^15^N_fix_). N content and mineral mass of gorse seeds were measured (n= 10 seeds), as well as the mean root:shoot ratio of gorse seedlings corresponding to the C1 and C2 size classes (n= 13 seedlings; unpublished data). Then, from the δ^15^N_seed_, we calculated the quantities of ^15^N and ^14^N in the shoot of the seedling that came from the seed. For each sample, we calculated a new isotopic ratio [^15^N]/[^14^N] by subtracting the nitrogen from the seed, and the values of δ^15^N_leg_ and %N_dfa_ were corrected accordingly.

*Analysis of the response of symbiotic nitrogen fixation to fertilisation***.**

The %N_dfa_ values were normalised with the logit transformation following Warton and Hui [9]. Type III ANOVAs for non-orthogonal samples (because of missing values) were performed to test the effect of the year, size class and fertilisation treatment on %N_dfa_ and N contents. Non parametric multiple comparisons of means were made ('nparcomp' R package) for differences in P content because of the small number of observations per class.

*Results and comments about the %N_dfa_, N and P contents.*

The sampling year influenced the measured %N_dfa_ of the young gorse plants (Table 3, main text). In addition, we observed a mean increase of 20% in the %N_dfa_ of fertilised areas in 2010. Conversely, %N_dfa_ was 10 % lower in case of fertilisation than in control areas in 2011. However, these intra-annual differences were only marginally significant. No difference was observed between the size classes.

Regarding N content in the plant tissues, we observed in 2011 a significant decrease in the N content with individual plant size (Table A). In both years, we found an increase in the P content with the size of the individuals (Table A). This increase was significant for the whole sample, and only in 2010 regarding intra-annual analyses. Moreover, we found significantly higher P content values for the whole sample in fertilised areas (P<0.05), but not in intra-annual analyses.

Surprisingly, we found a significant increase of the %N_dfa_ in 2011 compared to the results of 2010 (Table 3, main text). Gorse seedlings showed a low growth rate until they reached the size threshold for successful recruitment (Figure A in S1 File). Consequently, small gorse plants sampled in 2011 may have emerged in 2010, when the majority of the seedlings appeared. Thus, the measurements made in 2010 may correspond strictly to the initial period of the establishment of the symbiosis, while in 2011, the measurements may correspond to symbiotic nitrogen fixation over a longer lapse of time, during which the symbiosis was functionally mature. This may explain the observed difference. The measurements of the %N_dfa_ in 2010 were smaller than those found in the literature for mature gorse plants in the region [4] while the measurements in 2011 were consistent with the literature. This comparison supports our interpretation.

In *2010,* we found a marginally significant positive effect of fertilisation on the %N_dfa._ Phosphorus fertilisation may have favoured the establishment of the symbiosis slightly, and a larger sample might have enabled a more straightforward conclusion. Following McLaughlin et al. [1], the overall increase in P content of fertilised areas is consistent with this interpretation.

In *2011*, the fixation rate of fertilised areas seemed lower than in the control areas. However, this difference was slight, the values of %N_dfa_ being close in the different treatments and they were consistent with the values published for mature gorse plants [6]. Furthermore, N content in the small plants may be high because of optimal symbiotic nitrogen fixation in 2011 [4]. The decrease in N content with the size of the plants may result from a dilution effect of the nitrogen mineral mass in the overall plant biomass [10]. The increase in the P content with the size of the plants suggests an increase of the capacity of phosphorus uptake with the development of root system of the plants.

On the whole, the effect of fertilisation on the fixation of nitrogen by the seedlings was low. This is consistent with the low limitation of nitrogen fixation by phosphorus availability for most mature legumes [11]. It implies that the main positive effect of phosphorus on the recruitment of new gorse plants is an improved seedling growth (see main text).

| N (mg.g^-1^) | | | | | | | | | |  | P (mg.g^-1^) | | | | | | | | | |
| --- | --- | --- | --- | --- | --- | --- | --- | --- | --- | --- | --- | --- | --- | --- | --- | --- | --- | --- | --- | --- |
| Year (n.s) | | | |  | Size class (n.s) | | | | |  | Year (n.s) | | | |  | Size class (**) | | | | |
|  | Mean | ± SE | n |  |  | Mean | ± SE |  | n |  |  | Mean | ± SE | n |  |  | Mean | ± SE |  | n |
| 2010 | 15.7 | ± 0.5 | 52 |  | C1 | 14.4 | ± 0.9 |  | 17 |  | 2010 | 0.61 | ± 0.07 | 26 |  | C1 | 0.11 | ± 0.02 | A | 2 |
|  |  |  |  |  | C2 | 16.5 | ± 0.8 |  | 17 |  |  |  |  |  |  | C2 | 0.43 | ± 0.09 | B | 6 |
|  |  |  |  |  | C3 | 16.2 | ± 1.0 |  | 18 |  |  |  |  |  |  | C3 | 0.73 | ± 0.08 | B | 18 |
| 2011 | 15.9 | ± 0.4 | 70 |  | C1 | 18.5 | ± 1.0 | b | 16 |  | 2011 | 0.57 | ± 0.03 | 61 |  | C1 | 0.44 | ± 0.06 |  | 8 |
|  |  |  |  |  | C2 | 16.6 | ± 1.0 | ab | 18 |  |  |  |  |  |  | C2 | 0.59 | ± 0.07 |  | 17 |
|  |  |  |  |  | C3 | 14.7 | ± 0.5 | a | 18 |  |  |  |  |  |  | C3 | 0.57 | ± 0.05 |  | 18 |
|  |  |  |  |  | C4 | 14.1 | ± 0.6 | a | 18 |  |  |  |  |  |  | C4 | 0.62 | ± 0.06 |  | 18 |

**Table A.** Variations of the rate of symbiotic nitrogen fixation (%N_dfa_), N and P content with the year of study, fertilization and size class.

N and P content measured per year, and depending on the size class of each year. Information given in brackets indicates the result of the ANOVA in the whole sample (**, P<0,01). Different uppercase letters indicate significant differences between classes performing within-year tests (P<0.05). Different lowercase letters indicate only marginal differences (P<0.1). The number of values of P content is reduced for the small size classes, because samples were pooled to have enough material for analysis (at least 300 mg).

**References**

1. McLaughlin MJ, Malik KA, Memon KS, Idris M (6-10/03/89) The role of phosphorus in nitrogen fixation in upland crops. In: (IRRI) IRRI, editor. pp. 295–305.

2. Oberson A, Nanzer S, Bosshard C, Dubois D, Mader P, et al. (2007) Symbiotic N-2 fixation by soybean in organic and conventional cropping systems estimated by N-15 dilution and N-15 natural abundance. Plant Soil 290: 69–83.

3. Handley LL, Raven JA (1992) The Use Of Natural Abundance Of Nitrogen Isotopes In Plant Physiology And Ecology. Plant Cell Environ 15: 965–985.

4. Cavard X, Augusto L, Saur E, Trichet P (2007) Field effect of P fertilization on N-2 fixation rate of Ulex europaeus. Ann For Sci 64: 875–881.

5. Hogberg P (1997) Tansley review No 95 - N-15 natural abundance in soil-plant systems. New Phytol 137: 179–203.

6. Augusto L, Crampon N, Saur E, Bakker MR, Pellerin S, et al. (2005) High rates of nitrogen fixation of Ulex species in the understory of maritime pine stands and the potential effect of phosphorus fertilization. Can J For Res 35: 1183–1192.

7. Van Veldhoven PP, Mannaerts GP (1987) Inorganic and organic phosphate measurements in the nanomolar range. Anal Biochem 161: 45–48. doi:10.1016/0003-2697(87)90649-x.

8. Drake DC (2011) Invasive legumes fix N-2 at high rates in riparian areas of an N-saturated, agricultural catchment. J Ecol 99: 515–523. doi:10.1111/j.1365-2745.2010.01787.x.

9. Warton DI, Hui FKC (2011) The arcsine is asinine: the analysis of proportions in ecology. Ecology 92: 3–10. doi:10.1890/10-0340.1.

10. Augusto L, Bakker MR, De Lavaissiere C, Jordan-Meille L, Saur E (2009) Estimation of nutrient content of woody plants using allometric relationships: quantifying the difference between concentration values from the literature and actuals. Forestry 82: 463–477.

11. Augusto L, Delerue F, Gallet-Budynek A, Achat DL (2013) Global assessment of limitation to symbiotic nitrogen fixation by phosphorus availability in terrestrial ecosystems using a meta-analysis approach. Glob Biogeochem Cycles 27: 804–815. doi:10.1002/gbc.20069.
