# Supplementary material for: Weak Evidence of Regeneration Habitat but Strong Evidence of Regeneration Niche for a Leguminous Shrub
Source: PLoS One. 2015 Jun 22;10(6):e0130886. doi: 10.1371/journal.pone.0130886 (PMC4476804; doi:10.1371/journal.pone.0130886)
Supplement: S3 File — (DOCX) [file pone.0130886.s003.docx]

**S3 File .** Supplementary results regarding the frequencies of seedling emergence and the different micro-topographic positions.

The relationships between seedlings emergence and micro-topographic positions were analysed with mixed logistic regression. Results are given in the Figure A and Figure B for 2010 and 2011 respectively (in 2011, the analysis was done only for emergences that occurred in spring because new seedlings rarely emerged later (see Figure 1 in the main text)).

In Figure A and B, for each of the five topographic positions, the difference between the proportion of microsites where seedling emergence occurred and the proportion observed for all microsites is represented with the different bar plots. At each date, these differences were standardised to allow comparisons of the response between different censuses with large variations of the observed numbers of emergences. (see the Figure 4b in the main text regarding the response in pits).


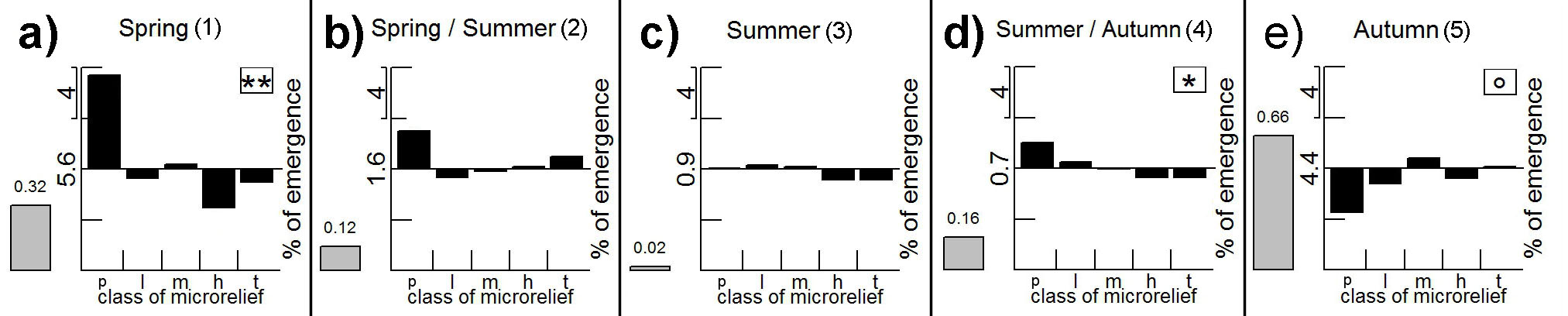


**Figure A**. Proportion of microsites where emergence of new seedlings was observed in the five micro-topographic positions during the five surveys made in 2010 for sown areas (a to e).

Grey rectangles on the left of the panels indicate the seasonal water availability index (SWAI index) during the interval before monitoring. Topographic classes of microsites are indicated on the x axis (p: pit; l: low; m: medium; h: high; t: top).

Symbols within the frame in the top righthand corner indicate the results of the analysis of deviance table (**, P<0.01; *, P<0.05; °, P<0,1).

**Figure B**. Proportion of microsites where emergence of new seedlings was observed in the five micro-topographic positions during two censuses made in 2011, for sown areas (a,b)

The SWAI index was not available for the interval before the first monitoring of 2011. See Figure A for explanations of the symbols and legends. **
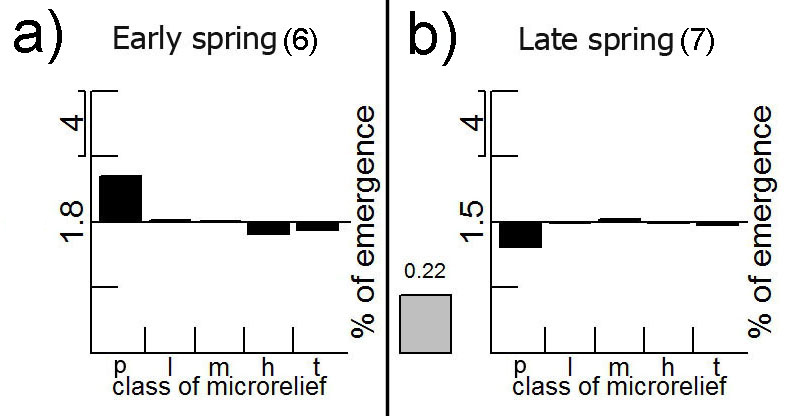
**
